# A simple, efficient, and rapid method for dye removal from wastewater using an IDA-GO@Fe_3_O_4_ magnetic nanocomposite†

**DOI:** 10.1039/d4ra04555f

**Published:** 2024-09-02

**Authors:** Amir Abdolmaleki, Zahra Mohamadi, Zahra Bazyar

**Affiliations:** a Department of Chemistry, College of Sciences, Shiraz University Shiraz 71467-13565 Iran abdolmaleki@shirazu.ac.ir abdolmaleki@iut.ac.ir +98-71-3613-7310 +98-71-3613-7187; b Department of Chemistry, Isfahan University of Technology Isfahan 84156-83111 Iran; c Department of Chemistry, Behbahan Khatam Alanbia University of Technology Behbahan 63616-63973 Iran

## Abstract

With the rapid advancement of the dye and textile industry, there has been increasing concern regarding the contamination of wastewater with dyes and its potential influence on human health. Therefore, the removal of dye pollutants from wastewater has become a matter of significant importance. In this study, a magnetically responsive iminodiacetic acid-functionalized graphene oxide (IDA-GO@Fe_3_O_4_) nanocomposite was utilized for the adsorption of both cationic and anionic dyes. The IDA-GO@Fe_3_O_4_ nanocomposite was synthesized and thoroughly characterized using several analytical techniques such as XRD, SEM, FT-IR, TGA and BET analysis. The prepared magnetic nanocomposite had a much higher thermal stability than pure graphene oxide. The negatively charged surface of the IDA-GO@Fe_3_O_4_ magnetic nanocomposite made it an excellent candidate for removing cationic dyes. The effects of various factors, including pH, initial concentration (isotherms), amount of adsorbent, and contact time (kinetics), on adsorption efficiency were investigated. The optimal conditions for the removal of methylene blue were determined to be 0.005 g of adsorbent, a pH of 10, and a contact time of 160 minutes. In contrast, the optimal conditions for the removal of methylene orange were found to be 0.005 g of adsorbent, a pH of 2, and a contact time of 120 minutes. Experimental data shows that the adsorption capacity for MB is reported as 437.10 mg g^−1^ at pH 10, while for MO it is 165.65 mg g^−1^ at pH 2. Furthermore, the adsorption process followed a pseudo-second-order kinetic model and Langmuir isotherm model. The proposed adsorption mechanism of MB and MO dyes onto the IDA-GO@Fe_3_O_4_ nanocomposite involve various interactions, such as electrostatic interactions, H-bonding, n–π, and π–π interactions. Importantly, the IDA-GO@Fe_3_O_4_ nanocomposite exhibited outstanding recyclability, retaining its effectiveness even after five successive cycles for MB. This suggests a straightforward method for developing high-performance IDA-GO@Fe_3_O_4_ magnetic nanocomposites for efficient wastewater purification and environmental remediation applications.

## Abstract 2

With the rapid advancement of the dye and textile industry, there has been increasing concern regarding the contamination of wastewater with dyes and its potential influence on human health.

## Graphical abstract

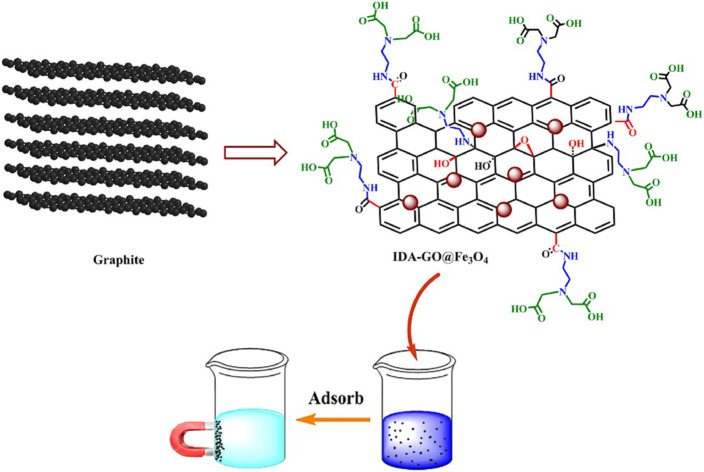

## 1. Introduction

There has been significant attention paid in recent years to the issue of organic water contamination.^1^ Specifically, treating dye-containing wastewater has become an important focus.^2^ Discharging organic dyes into wastewater can reduce sunlight penetration, diminishing aquatic plant photosynthesis.^3–5^ Furthermore, certain synthetic dyes have detrimental health effects, causing severe problems.^6,7^

Many techniques have been developed to eliminate dye pollutants from wastewater,^8–11^ coagulation/flocculation,^12^ zonation,^13,14^ membrane filtration,^15^ chemical oxidation,^16^ adsorption,^17^ and biological wastewater treatment involving microorganisms.^18^ Each approach has unique advantages depending on the wastewater characteristics.

Among these techniques, adsorption using solid adsorbents has gained traction as an effective, simple, and cost-efficient dye treatment method for industrial wastewater.^19–21^ Adsorption involves dye molecules attaching to adsorbent surfaces, removing them from water. This approach is favored for its simplicity, efficiency, and affordability, making it widely used industrially for wastewater treatment.^3,22,23^

The removal of dyes from different wastewaters has been achieved using a variety of adsorbents, including activated carbon, carbon nanotubes, nanoporous silica, and clays.^24–27^ However, these adsorbents often have limited capacities and can potentially contaminate the environment if not fully recovered.^24,26^ These drawbacks restrict their widespread wastewater application. Developing new adsorbents with high capacities and easy separability is crucial to address these challenges, improving wastewater treatment sustainability and efficacy.^27^

Oxidized graphite forms graphene oxide (GO), a two-dimensional carbon nanosheet. Oxygen functional groups for example carboxyl, carbonyl, epoxy, and hydroxyl are abundant in the honeycomb lattice structure of this material.^28–30^

These groups impart GO with hydrophilicity, reactivity, and aqueous dispersibility, enabling interactions with various substances.^31,32^ However, small GO nanosheet size hinders separation from water using centrifugation or filtration.^28,33^ Their high surface area-to-volume ratio complicates recovery using conventional techniques. Alternative approaches are needed to enable efficient GO nanosheet separation and recovery from aqueous systems.^34–36^

Utilizing magnetic nanoparticles can allow easy,^37–39^ rapid separation by using external magnetic fields, thus addressing this problem.^40,41^ This eliminates the need for centrifugation or filtration. The adsorbent gains magnetic properties *via* nanoparticle addition, enabling efficient recovery and reuse.^42,43^ This simplifies separation and improves wastewater treatment efficiency.

In this study, IDA-GO@Fe_3_O_4_ was synthesized for effective dye elimination from wastewater. The synthesis method involved reacting graphene oxide with ethylenediamine and chloroacetic acid to introduce iminodiacetic acid groups onto the graphene oxide surface. This modification improved the dispersion and adsorption properties of the material. Subsequently, iron oxide nanoparticles were incorporated into the modified graphene oxide, resulting in the formation of magnetic nanocomposites. The synthesized IDA-GO@Fe_3_O_4_ magnetic nanocomposites were then tested as adsorbents for the removal of methylene blue and methyl orange dyes from aqueous solutions. The results showed that the nanocomposites exhibited excellent adsorption capacity for both dyes. The kinetic and equilibrium data obtained from the adsorption process were found to conform to the pseudo second-order model, indicating a chemisorption mechanism. These findings highlight the significant potential of magnetic IDA-GO@Fe_3_O_4_ nanocomposites as highly efficient sorbent materials for the removal of dyes from wastewater. The magnetic properties of the nanocomposites enable easy separation from the treated water, making them suitable for practical applications in dye wastewater treatment.

## 2. Results and discussion

### 2.1. Characterization of GO, EDA-GO, IDA-GO and IDA-GO@Fe_3_O_4_

**Fig. 1:**
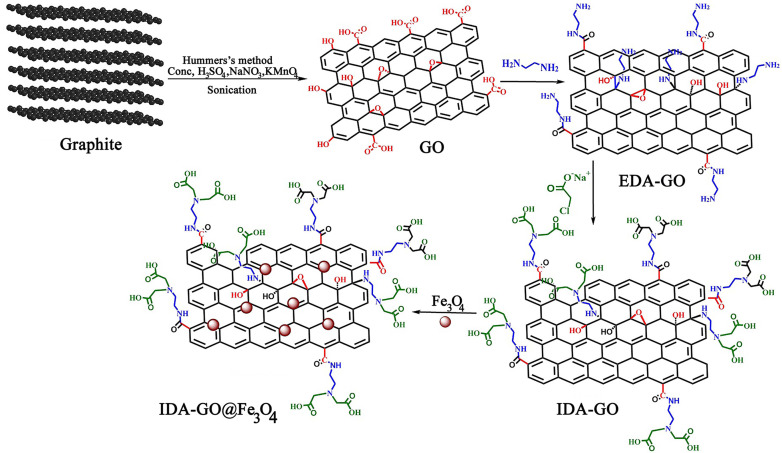
Synthesis of IDA-GO@Fe_3_O_4_ magnetic nanocomposite.

IDA-GO@Fe_3_O_4_ magnetic nanocomposite, which can serve as a magnetically separable adsorbent for water treatment, is illustrated in Fig. 1. As a result of the Hummers' method, graphene oxide (GO) was first produced by oxidizing graphite, which includes carboxylic acids, hydroxyls, and epoxides as oxygen functional groups. Next, the GO surface was functionalized with ethylenediamine (EDA) to attach amine groups *via* nucleophilic substitution of the epoxide and hydroxyl groups. Sodium chloroacetate then underwent a carboxymethylation reaction with the amine groups on EDA-GO. Finally, Fe_3_O_4_ magnetic nanoparticles were deposited on the graphene sheets in an alkaline environment. FTIR spectroscopy, Raman spectra, TGA and zeta potential of GO, EDA-GO, and IDA-GO were reported by our group previously (ESI†).^2^

**Fig. 2:**
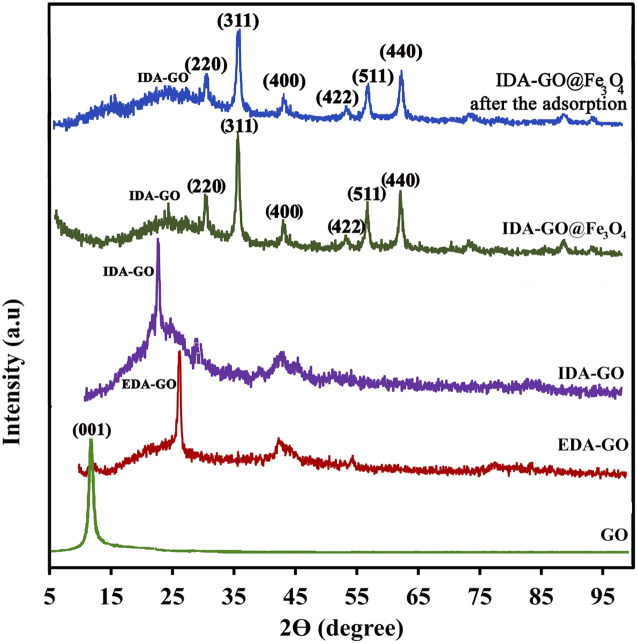
XRD pattern of GO, EDA-GO, IDA-GO, IDA-GO@Fe_3_O_4_ magnetic nanocomposite and IDA-GO@Fe_3_O_4_ magnetic nanocomposite after the adsorption.

The X-ray diffraction (XRD) patterns of graphene oxide (GO), ethylenediamine-functionalized graphene oxide (EDA-GO), and isothiocyanate-functionalized graphene oxide (IDA-GO) have been previously reported by our research group.^2^ The XRD patterns for IDA-GO@Fe_3_O_4_ and reused catalyst samples are illustrated in Fig. 2. Notably, the XRD pattern of GO displays a prominent diffraction peak at approximately 2*θ* = 10.6°. The emergence of a peak at 2*θ* = 26.5° in the EDA-GO spectrum indicates the successful attachment of ethylenediamine to the GO sheets. This shift in peak position suggests that ethylenediamine functions as both a linking agent and a reducing agent, facilitating the reduction of GO. Following the reaction with sodium chloroacetate, the peak associated with the functionalized graphene oxide shifts to a lower 2*θ* value of 22.4°, signifying a partial increase in the interlayer spacing of the graphene sheets. The presence of acetic acid groups on the graphene oxide layers contributes to this increase in interlayer spacing, resulting in an expansion of the inter planar distance. The peaks observed at 30.1°, 35.6°, 43.1°, 53.2°, 57.5°, and 62.8° correspond to the (220), (311), (400), (422), (511), and (440) planes, respectively, confirming the deposition of Fe_3_O_4_ nanoparticles onto the graphene sheets. The average particle sizes of IDA-GO@Fe_3_O_4_, as calculated using Scherrer's formula, range from approximately 40 nm, suggesting that the crystal structure of Fe_3_O_4_ remains unchanged following the modification of IDA-GO.

**Fig. 3:**
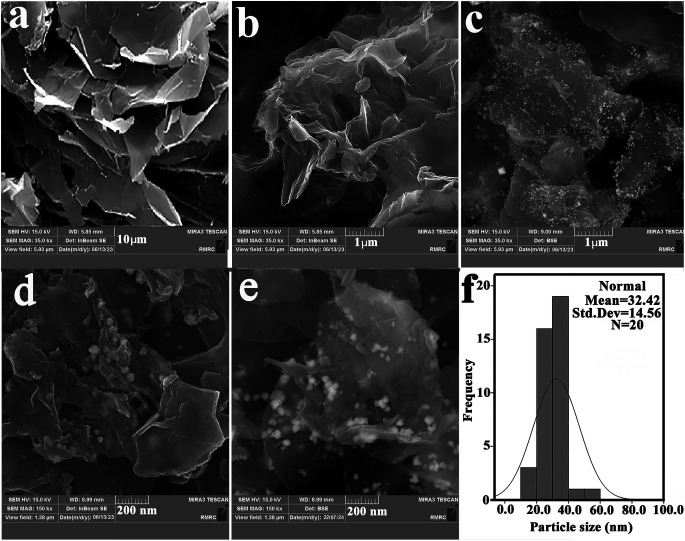
SEM images of graphite flake (a), IDA-GO (b) and IDA-GO@Fe_3_O_4_ magnetic nanocomposite (c and d), IDA-GO@Fe_3_O_4_ magnetic nanocomposite after the adsorption (e and f) size-distribution graph for IDA-GO@Fe_3_O_4_.

Also, the comparison of the XRD pattern of the recovered IDA-GO@Fe_3_O_4_ magnetic nanocomposite with that of the fresh one indicates that the density of these peaks was decreased after MB adsorption, which was characterized by peak at 35.6° morphology and microstructure of synthesized samples were analyzed using scanning electron microscopy (SEM). Surfaces are imaged using SEM using a focused beam of electrons. SEM images of graphite flake, IDA-GO and IDA-GO@Fe_3_O_4_ magnetic nanocomposite are shown in Fig. 3. The IDA-GO exhibits a layered structure with wrinkled and folded features. The high surface area of these thin nanosheets with edge planes and defects can facilitate enhanced interaction with target analytes including dyes (Fig. 3b). In contrast, Fig. 3c and d show the IDA-GO@Fe_3_O_4_ magnetic nanocomposite, where Fe_3_O_4_ nanoparticles are clearly seen uniformly coating the IDA-GO support. The incorporation of Fe_3_O_4_ provides magnetic functionality while the IDA-GO nanosheet structure offers a high surface area substrate for analyte adsorption and removal. Also, the average diameters of the IDA-GO@Fe_3_O_4_ magnetic nanocomposite was 32 nm.

The SEM results are in agreement with observations from XRD. Summary, SEM provided valuable insights into the structural and morphological characteristics of IDA-GO after modification with Fe_3_O_4_ nanoparticles. Also, the comparison of the SEM image of the recovered IDA-GO@Fe_3_O_4_ magnetic nanocomposite with that of the fresh one indicates that the morphology of the catalyst has not changed after recovery (Fig. 3e).

The specific surface area and pore size are critical factors influencing the adsorption properties of an adsorbent. As presented in Table 1, the specific surface area and average pore diameter of the IDA-GO@Fe_3_O_4_ magnetic nanocomposite are greater than IDA-GO, GO and Fe_3_O_4_. This increase in total surface area is correlated with a significant enhancement in the adsorption capacity of the IDA-GO@Fe_3_O_4_ magnetic nanocomposite. These findings can be attributed to the formation and anchoring of Fe_3_O_4_ nanoparticles (NPs) and functional groups iminodiacetic acid groups on the surface of GO, which reduces the aggregation of Fe_3_O_4_ and minimizes the stacking of GO sheets.

**Table 1:** BET specific surface areas of Fe_3_O_4_, GO, IDA-GO and IDA-GO@Fe_3_O_4_ magnetic nanocomposite

Materials	Specific surface area (m^2^ g^−1^)	Pore volume (cm^3^ g^−1^)
Fe_3_O_4_	89.45	0.19
GO	128.65	0.25
IDA-GO	179.23	0.28
IDA-GO@Fe_3_O_4_	201.28	0.32

These results could be explained by Fe_3_O_4_ NPs were formed and anchored on the surface of IDA-GO, decreasing the aggregation of Fe_3_O_4_ and stacking of GO sheets.

## 3. Adsorption studies

The synthesized IDA-GO@Fe_3_O_4_ magnetic nanocomposite possesses amine, amide, carboxylic acid and hydroxyl functional groups that can engage in pollutant adsorption. The surface acidity and negative zeta potential also promote adsorption of cationic dyes. To elucidate the adsorption mechanisms comprehensively, pH, Langmuir and Freundlich isotherms, and adsorption kinetics were examined. By functionalizing GO, pollutant adsorption can be enhanced. Cationic and anionic dyes were selected as model adsorbents due to their prevalence in textile wastewaters. Systematic experiments provide insights into the relationships between adsorbent properties and dye adsorption performance. Overall, this work evaluates the utility of novel functionalized GO nanocomposites for sustainable treatment of cationic and anionic dyes containing wastewaters.

### 3.1. Effect of solution pH

**Fig. 4:**
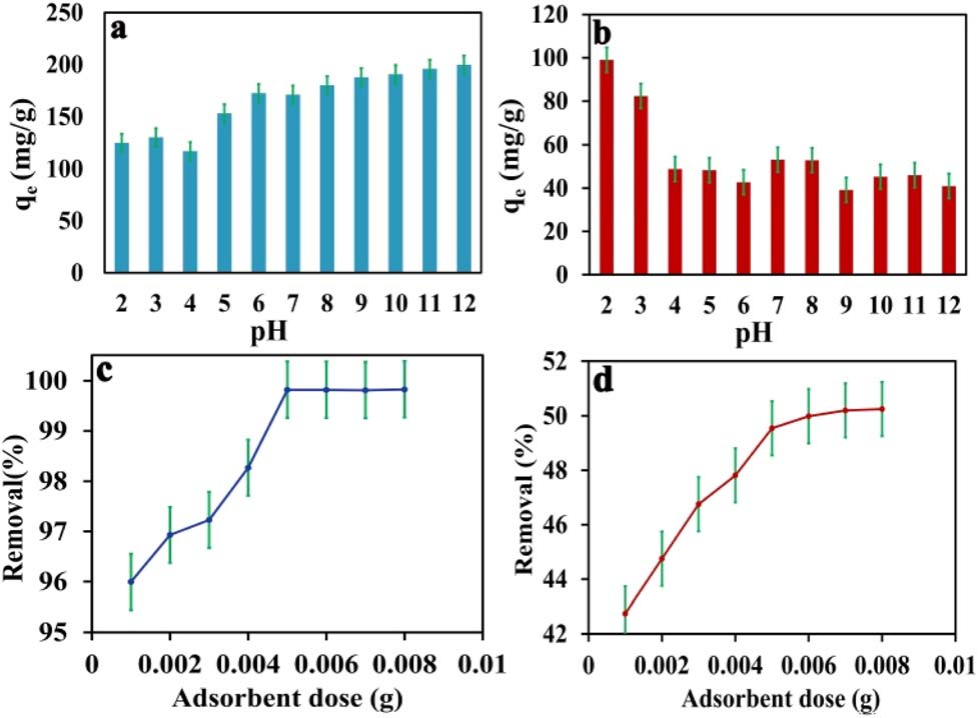
pH effects on MB and MO adsorption capacity (a) and (b), adsorbent dose effect on MB and MO adsorption capacity (c) and (d). The error bar represents the standard error (*n* = 4).

The study focused on investigating the influence of electrostatic interactions on dye adsorption in wastewater by adjusting the pH within the range of 2 to 12. The adjustment of pH has a significant impact on the surface charge of the adsorbent material and the ionization of the dye molecules. The results demonstrated that the optimal pH level for the adsorption of methylene blue (MB) was found to be 10, leading to enhanced removal of cationic methylene blue dye, as illustrated in Fig. 4. At low pH levels, the surface of IDA-GO@Fe_3_O_4_ magnetic nanocomposite exhibited a positive charged, resulting in the competitive binding of hydronium ions over cationic dyes. However, IDA-GO@Fe_3_O_4_ magnetic nanocomposite, which contains carboxyl, epoxy, and hydroxyl groups, exhibited a negative surface charge across the pH range of 2–12. The investigation of pH manipulation within this range aimed to understand its influence on dye adsorption in wastewater, with a specific focusing on the electrostatic interactions. The study revealed that increasing the pH level enhanced the surface negativity of the adsorbent material, thus promoting the adsorption of cationic methylene blue (MB) done electrostatic attraction. Conversely, the removal of the anionic dye methyl orange (MO) decreased at higher pH values due to deprotonation, which increased the negative surface charge density of IDA-GO@Fe_3_O_4_, and resulted in intensified electrostatic repulsion. The optimal pH for the adsorption of MB was found to be 10, while for MO, it was achieved at pH 2. In conclusion, the pH-dependent surface charge and ionization of the adsorbed dye molecules play a critical role in governing dye adsorption through electrostatic effects. Cationic dyes tend to favor higher pH levels, where the adsorbent surface is negatively charged, while anionic dyes exhibit higher removal efficiency at lower pH levels.

### 3.2. Effect of adsorbent dose

The dosage of the adsorbent was varied within the range of 0.001 to 0.008 g, while maintaining constant values for all other experimental parameters, including pH, initial concentration, and contact time.

Fig. 4c and d illustrate the relationship between adsorbent dosage and the percentage removal of methylene blue (MB) and methylene orange (MO). It is observed that as the adsorbent dosage increases, the percentage removal of both dyes increases, reaching a maximum at a dose of 0.005 g. This enhancement in dye removal can be attributed to the increased surface area of the adsorbent and the availability of additional sorption sites resulting from the higher dosage. However, beyond an adsorbent amount of 0.005 g, the increase in dye removal becomes negligible, as the concentration of dyes on the surface of the adsorbent approaches equilibrium with the concentration of dyes in the solution. Specifically, the percentage removal of MB increases from 95.8% to 99.9%, while the percentage removal of MO rises from 42.7% to 50.07% as the adsorbent dosage is increased from 0.001 g to 0.008 g, respectively (see Fig. 4c and d).

### 3.3. Isotherms of adsorption

An adsorption isotherm is formulated on the assumption of homogeneous surfaces for Langmuir adsorption.^44^ The equation is as follows:1
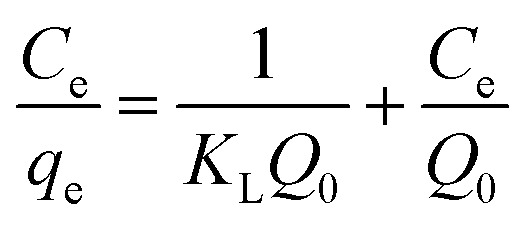


**Fig. 5:**
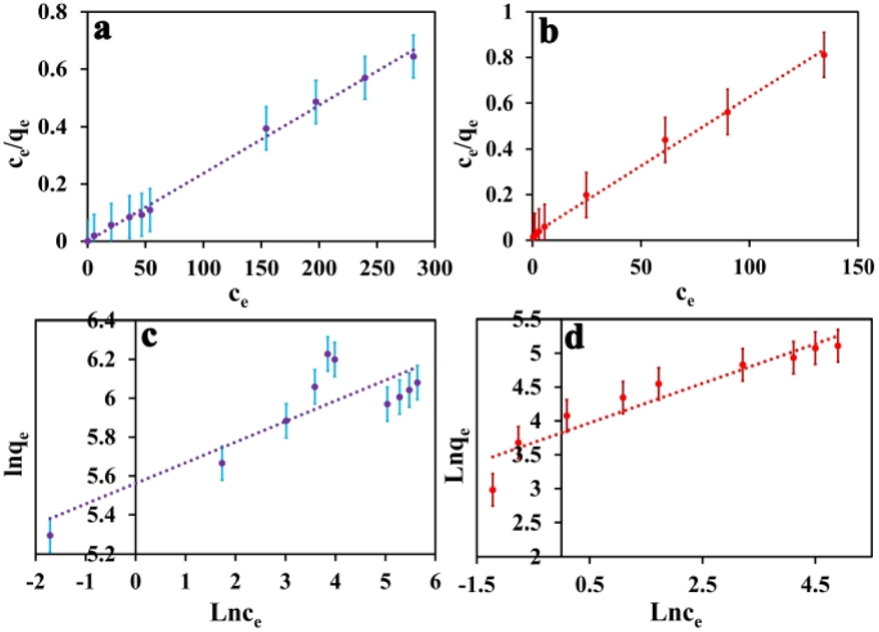
The Langmuir adsorption isotherm model of MB (a) and MO (b), as well as the Freundlich adsorption isotherm model of MB (c) and MO (d). The error bar represents the standard error (*n* = 4).

To achieve complete monolayer coverage, *Q*_0_ represents the maximum amount of dye that can be adsorbed per unit weight of the adsorbent. The Langmuir adsorption constant, *k*_L_, is expressed in units of L mg^−1^ and reflects the affinity of the adsorbent–adsorbate interaction. To assess whether the adsorption follows Langmuir behavior, a plot of *C*_e_/*q*_e_ (equilibrium concentration per amount adsorbed) *versus C*_e_ (equilibrium concentration) can be constructed. If the adsorption process adheres to the Langmuir model, this plot should yield a linear relationship. A Freundlich adsorption isotherm, however, does not assume a homogeneous surface and describes adsorption on heterogeneous surfaces. Freundlich's equation is as follows:2
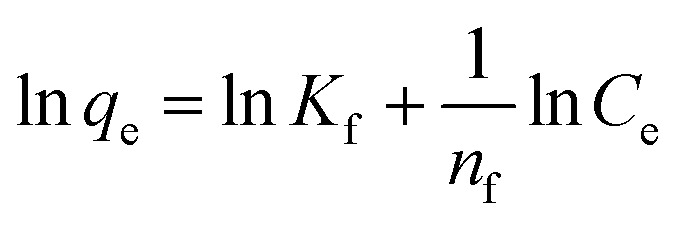
where characteristic constant is given by *k*_f_ and *n*_f_. These two isotherms, commonly utilized, serve to fit experimental adsorption data and offer insights into the adsorption process on IDA-GO@Fe_3_O_4_ magnetic nanocomposite. Methylene blue (MB) and methyl orange (MO) exhibit different adsorption isotherms for IDA-GO@Fe_3_O_4_ magnetic nanocomposite. Comparing the Langmuir model to the Freundlich model demonstrates that the Langmuir model provides a better fit to the data. This suggests there are relatively homogenous binding sites on the IDA-GO@Fe_3_O_4_ surface, even with multiple surface functional groups. A summary of the Langmuir and Freundlich isotherm constants is presented in Table S1.† In the Langmuir model, MO had a maximum adsorption capacity of 165.65 mg g^−1^, while MB had a maximum adsorption capacity of 437.10 mg g^−1^. Observations of high equilibrium concentrations confirmed Langmuir's monolayer coverage assumption on a homogeneous surface (Fig. 5). In summary, Langmuir's model better described the adsorption isotherms, indicating that the nanocomposite surface had homogeneous binding sites.

### 3.4. Kinetics of adsorption

**Fig. 6:**
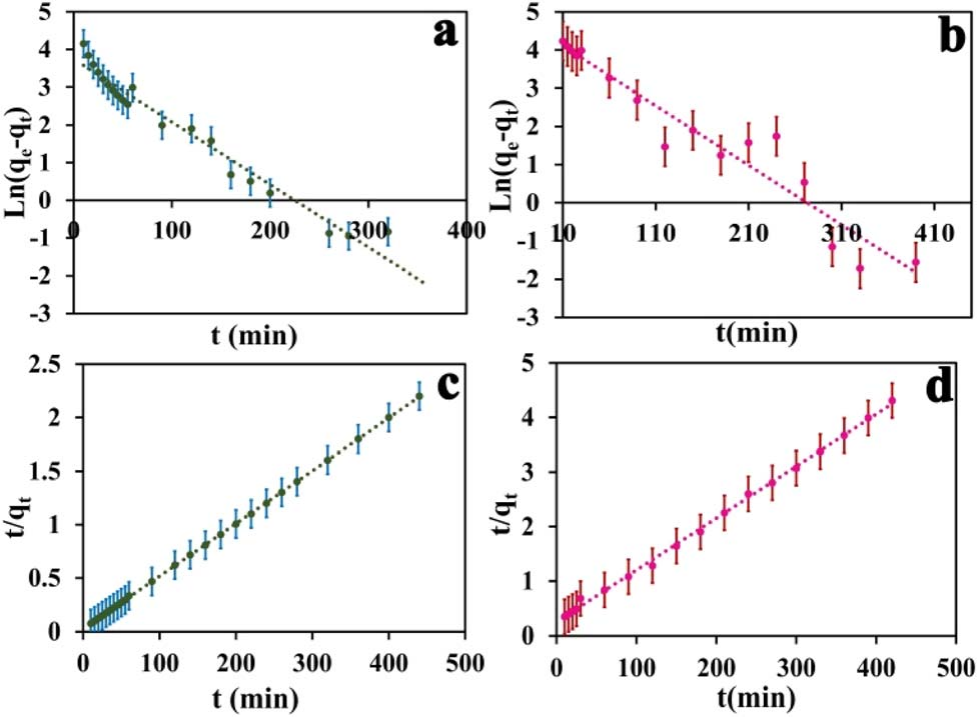
MB dye pseudo-first-order kinetic curve (a), MO dye pseudo-first-order kinetic curve (b). MB dye pseudo-second-order kinetic curve (c), MO dye pseudo-second-order kinetic curve (d). The error bar represents the standard error (*n* = 4).

In the context of the adsorption process, there are four distinct steps involved: external mass transfer in the bulk liquid phase, boundary layer diffusion, intra-particle mass transfer within particles, and sorption on active sites.^45^ There are two key steps in adsorption kinetics-external mass transfer from the solution to the surface of the adsorbent, and intraparticle diffusion inside the pores of the adsorbent. These steps control the rate of adsorbate transport and availability to active binding sites on the adsorbent. As seen in Fig. 6, the equilibrium adsorption capacity for MB and MO shows a rapid increase within the first 120 and 160 minutes, respectively. Subsequently, the adsorption capacity gradually increases until 440 minutes and eventually reaches a saturation point. In the initial rapid phase, there are many vacant sites on the adsorbent, which fill up as adsorption proceeds. Analyzing the adsorption kinetics was done using pseudo-first-order and pseudo-second-order kinetic models. These models describe the rate and mechanism of adsorbate binding onto solid adsorbent surfaces. In summary, the kinetics are governed by mass transfer effects and adsorbent site availability. Modeling of the kinetics of adsorption can provide quantitative insight into the adsorption mechanisms and steps that control their rate. The equation for first order rate is as follows:

3 ln(*q*_e_ − *q*_*t*_) = Ln(*q*_e_) − *K*_1_*t*

The equilibrium amount of adsorbate is *q*_e_, the equilibrium amount of adsorbate is *q*_*t*_, *K*_1_ is the first order reaction rate constant, and *t* is the adsorption time. There is little agreement between experimental and calculated values of equilibrium adsorption (*q*_e_), and correlation coefficients (*R*_2_) are relatively modest. This suggests that the first-order rate equation may not accurately describe the adsorption process. As such, alternative models or equations should be explored to enhance the correlation between experimental and calculated results and better reflect the kinetics of adsorption. Following is an illustration of the pseudo-second-order kinetic model:4
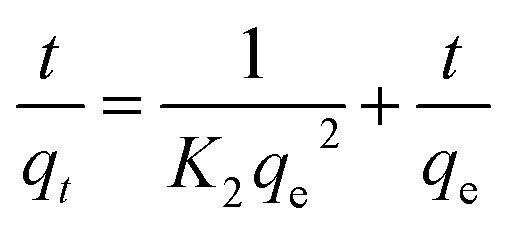


In this case, *K*_2_ refers to the pseudo-second-order adsorption rate constant. Experimental and calculated *q*_e_ values agree well across diverse initial concentrations in linear plots of *t*/*q*_*t*_*versus* time (Fig. 6). The pseudo-second-order kinetic model clearly proves to be an appropriate approach for elucidating the adsorption outcomes pertaining to both anionic and cationic dyes (Table S2†) based on the correlation coefficient and the concordance between experimental and calculated adsorption capacities. Therefore, the pseudo-second-order kinetic model accurately describes the adsorption process for both dye types and offers a satisfactory fit to the experimental data.

### 3.5. The proposed adsorption mechanism

**Fig. 7:**
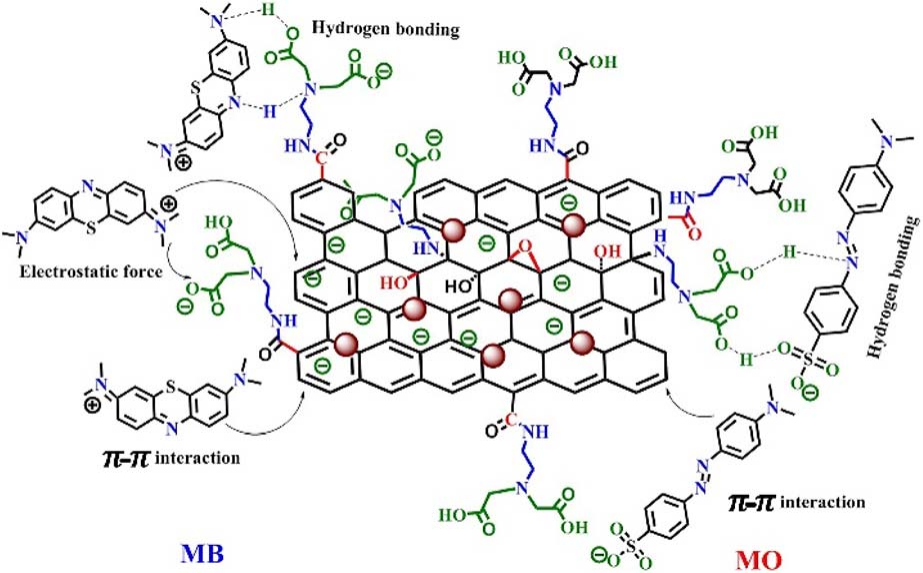
Different sorption mechanisms of IDA-GO@Fe_3_O_4_ for methylene blue and methyl orange.

Kinetic and isotherm studies indicate that both physical and chemical interactions play significant roles in the adsorption of MB onto the IDA-GO@Fe_3_O_4_ magnetic nanocomposite. Various potential adsorption mechanisms for the dyes on the IDA-GO@Fe_3_O_4_ magnetic nanocomposite are depicted in Fig. 7. Both MB and MO possess π-conjugated backbones, which likely facilitate their adsorption onto the IDA-GO@Fe_3_O_4_ magnetic nanocomposite through π–π stacking interactions between the carbon basal plane of graphene oxide (GO) and the aromatic rings of the dyes.

Additionally, both MB and MO can engage in hydrogen bonding with the numerous hydroxyl and amine groups present in the IDA-GO@Fe_3_O_4_ magnetic nanocomposite structure. Specifically, for MB, hydrogen bonds may form between the nitrogen or sulfur heteroatoms in its aromatic ring and the hydroxyl (OH) and amine (NH) groups of the IDA-GO@Fe_3_O_4_ magnetic nanocomposite, as illustrated in Fig. 7. In the case of MO, hydrogen bonding can occur *via* the oxygen atoms in the sulfonate group (–SO_3_) with the hydroxyl groups of the IDA-GO@Fe_3_O_4_ magnetic nanocomposite.

Furthermore, the surface of the IDA-GO@Fe_3_O_4_ magnetic nanocomposite carries a negative charge across a broad pH range, enabling it to adsorb MB through electrostatic interactions, which arise from attractions between species with opposite electrical charges. Moreover, the adsorption pathways for MB and MO *via* π–π interactions may exhibit differing strengths. MB features three adjacent, fused aromatic rings aligned in the same plane, while MO's two aromatic rings are separated by an N=N bond, resulting in out-of-plane bending. Consequently, the π–π interactions between MO and the aromatic rings of IDA-GO@Fe_3_O_4_ magnetic nanocomposite sheets are anticipated to be weaker than those involving MB. As a result, the adsorption capacity of IDA-GO@Fe_3_O_4_ magnetic nanocomposite for MB is significantly greater than that for MO.^28,45^

### 3.6. Comparison of adsorption capacity of the prepared GO, IDA-GO and IDA-GO@Fe_3_O_4_ magnetic nanocomposite adsorbents

The maximum adsorption capacity for the adsorption of MB by GO, IDA-GO and IDA-GO@Fe_3_O_4_ magnetic nanocomposite adsorbents were found to be 302.4, 385.2 and 437.10 mg g^−1^ at pH 10, respectively, the IDA-GO@Fe_3_O_4_ magnetic nanocomposite has higher adsorption capacity than GO and IDA-GO. The results clearly demonstrate that graphene modified with iminodiacetic acid exhibits superior performance in adsorbing methylene blue (MB) compared to methyl orange (MO). This can be attributed to the strong electrostatic interaction between the positively charged MB molecules and the negatively charged modified graphene. Also, as can be seen in Table 1, specific surface area and average pore diameter are higher for IDA-GO@Fe_3_O_4_ magnetic nanocomposite than GO, Fe_3_O_4_. It was observed that, the increase in total surface area resulted in a significant increase in the IDA-GO@Fe_3_O_4_ adsorption capacity.

## 4. Desorption and regeneration studies

**Fig. 8:**
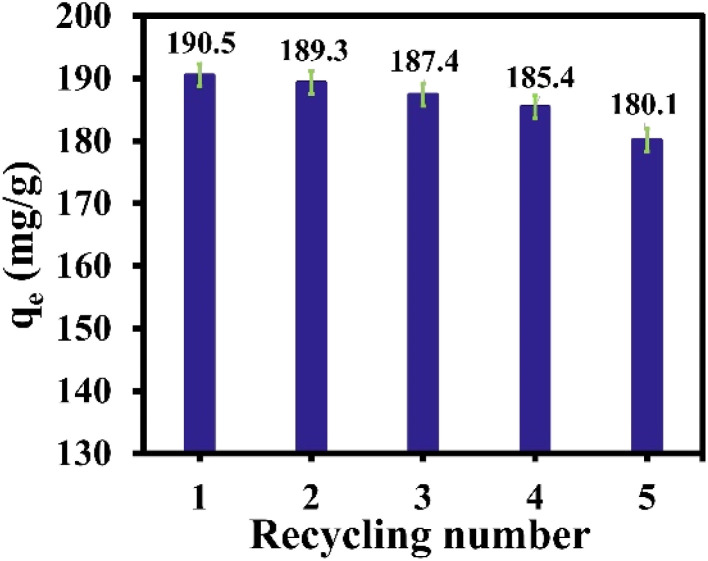
Recyclability of IDA-GO@Fe_3_O_4_ magnetic nanocomposite for MB. The error bar represents the standard error (*n* = 4).

The ability regeneration to regenerate the adsorbent is crucial for its practical application. In the present study, desorption studies were carried out with adsorbents such as water and ethanol for the IDA-GO@Fe_3_O_4_ magnetic nanocomposite. The results of 5 cycles of adsorption and desorption are shown in Fig. 8 indicating that the adsorption capacity for MB partially decreases. Thus, the prepared adsorbent can be easily recovered and used as an effective and powerful adsorbent multiple times without a decrease in performance.

The performance of IDA-GO@Fe_3_O_4_ magnetic nanocomposite in purifying MB and MO dyes wastewater were compared with previously published works, and the results are summarized in Table 2. The data presented in Table S3† clearly demonstrates the superior performance of the IDA-GO@Fe_3_O_4_ magnetic nanocomposite developed in this study.

**Table 2:** Comparison the performance of prepared IDA-GO@Fe_3_O_4_ magnetic nanocomposite with those reported in literature

Adsorbent	Dye	*q*_max_ (mg g^−1^)	Ref.
IDA-GO@Fe_3_O_4_	MB	437.10	This work
IDA-GO@Fe_3_O_4_	MO	165.65	This work
GO@CA beads	MB	396.85	29
UiO-66/GOCOOH@SA	MB	343.49	33
Fe_3_O_4_@GO@SA composite	MB	112	46
CA/MOF-DPC composite	MB	41.36	47
PT-GO composite	MB	256.58	48

Notably, at a higher dye concentration of 100 ppm, the IDA-GO@Fe_3_O_4_ magnetic nanocomposite exhibits remarkable potential for efficiently purifying dye wastewater, which is a feature that has been less documented for other nanocomposite.

## 5. Conclusions

This study successfully synthesized a new IDA-GO@Fe_3_O_4_ IDA-GO@Fe_3_O_4_ magnetic nanocomposite with effective adsorption capabilities for both cationic and anionic dyes in wastewater. The stepwise functionalization of graphene oxide, incorporating abundant iminodiacetic acid (IDA) groups along with Fe_3_O_4_ nanoparticles, resulted in improved dispersion stability and magnetic separability of the nanocomposite. The results clearly demonstrate that graphene modified with iminodiacetic acid exhibits superior performance in adsorbing methylene blue (MB) compared to methyl orange (MO). This can be attributed to the strong electrostatic interaction between the positively charged MB molecules and the negatively charged modified graphene. Experimental data shows that the adsorption capacity for MB is reported as 437.10 mg g^−1^ at pH 10, while for MO it is 165.65 mg g^−1^ at pH 2. Some mechanisms, counting hydrogen bonding, π–π interactions, and electrostatic interactions, contribute to the adsorption process between the dye and the adsorbent. Measurements of the zeta potential indicate that the prepared adsorbent maintains a negative electrical charge over a wide pH range. Hence, it is expected that the adsorbent would exhibit better performance in adsorbing cationic dyes compared to anionic dyes. The adsorption of MB increases with an increase in pH because of the higher concentration of negative charges on the adsorbent surface. Additionally, the planar structure of MB facilitates favorable π–π interactions with the graphene sheets, further enhancing its adsorption. On the other hand, the non-planar structure of MO and the electrostatic repulsion between the anionic dye and the graphene sheets lead to a reduction in the adsorption capacity. The adsorption process follows pseudo-second-order kinetics, with the rate being determined by mass transfer and the availability of adsorbent sites. Equilibrium data supports a homogeneous monolayer binding, fitting well with the Langmuir isotherm. Overall, this facile synthesis approach yields a magnetically recoverable adsorbent with high dye adsorption capacity, rapid kinetics, and the potential for recyclability. The IDA-GO@Fe_3_O_4_ nanocomposite demonstrates promising capabilities for the efficient and sustainable treatment of industrial wastewaters containing cationic dyes.

## 6. Experimental

### 6.1. Materials

Analytical-grade chemicals from Sigma-Aldrich were used in the study, including ethylenediamine, sodium chloroacetate, FeCl_3_·6H_2_O, FeCl_2_·4H_2_O, methylene blue, and methyl orange.^2^ Graphite flakes from Merck Company were also used. All chemicals were used as received without additional purification.

### 6.2. Synthesis of GO and GO functionalized with iminodiacetic acid (IDA-GO)

The graphene oxide (GO) and EDA-GO were synthesized as previously reported.^23^

### 6.3. Synthesis of graphene functionalized with magnetically responsive iminodiacetic acid (IDA-GO@Fe_3_O_4_)

Based on a modified protocol from,^49^ magnetic nanocomposites were synthesized. IDA-GO was ultrasonically dispersed in water for 30 minutes. A solution of FeCl_3_·6H_2_O (162 mg) and FeCl_2_·4H_2_O (60 mg) was gradually added to 4 mL water under N_2_ atmosphere over 2 hours. Afterward increasing the temperature to 90 °C, ammonia solution was incrementally added until pH 10 was reached. A precipitate was collected after reacting for 6 hours using an iron spatula, washed thoroughly in water, and dried.

### 6.4. Dyes adsorption measurement

The synthesized IDA-GO@Fe_3_O_4_ magnetic nanocomposite was used to conduct batch adsorption studies on methylene blue (MB) and methyl orange (MO). 5 mg of nanocomposite was added to 10 mL of dye solution with a known initial concentration. A shaker was used to facilitate adsorption of the mixture. A UV-vis spectrophotometer at 662 nm for MB and 500 nm for MO was used to measure the supernatant adsorbance at various time intervals. The final dye concentrations were calculated by correlating the adsorbance values with calibration curves. Equations were used to estimate adsorption capacity at time (*q*_*t*_, mg of dye per g of adsorbent) and removal efficiency:5
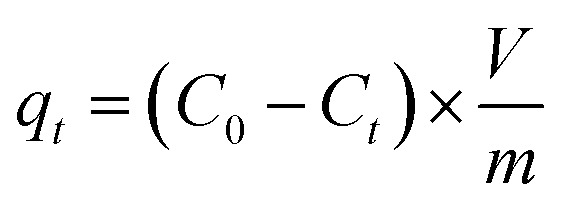

6 Removal efficiency (%): = (*C*_0_ − *C*_*t*_)/*C*_0_ × 100

*C*_*t*_ = concentration of the dye at time *t* (mg of dye per liter of solution), *C*_0_ = initial concentration of the dye at the beginning of the experiment (mg of dye per liter of solution), *V* = volume of the MB or MO solution (in liters), *m* = mass of the adsorbent (in grams).

These equations allow for determining the adsorption capacity at a specific time point, as well as the overall dye removal efficiency by the IDA-GO@Fe_3_O_4_ nanocomposite. The solution pH was adjusted to desired values using diluted NaOH to increase the pH (make more basic) and HCl to decrease the pH (make more acidic). Controlling the pH is crucial in many experiments as it can influence chemical reactions, solubility, and adsorption behavior of substances in solution.

### 6.5. Recycling and reusability of magnetic nanocomposite

Recovery studies of the IDA-GO@Fe_3_O_4_ magnetic nanocomposite for adsorption were conducted at room temperature. In each adsorption–desorption cycle, a specific amount of the IDA-GO@Fe_3_O_4_ magnetic nanocomposite was added to an aqueous solution of methylene blue (MB). After reaching equilibrium, the adsorbent was separated using a strong external magnetic field. The nanocomposite was then regenerated with ethanol for 2 hours and subsequently washed with an adequate volume of distilled water. The regenerated adsorbent was utilized in the subsequent cycle. This reusability study was performed over five cycles.

### 6.6. Statistical analysis

Data presented are the means from four independent experiments, and statistical analysis (error bars) was carried out using Excel 2019. Data represent the average of four independent experiments ±SD shown by error bars (Fig. 4–6 and 8). Microsoft excel was used to fit the experimental data to the selected models. Correlation coefficient (*R*_2_) tests were used to measure the appropriateness of the fit.

### 6.7. Characterization

The X-ray diffractometer (Philips X'PERT MPD) (Cu Kα, *λ* = 1.5418 Å) was used to obtain wide-angle X-ray diffraction (XRD) patterns. The morphology of the products was determined using a Leica Cambridge model S360, version V03.03. Scanning electron microscopy (SEM) was conducted at an accelerating voltage of 20 kV. A JASCO V-570 spectrophotometer (Tokyo, Japan) was used for UV-vis spectroscopy. The specific surface areas (SSABET; [m^2^ g^−1^]) of the catalyst were analyzed through nitrogen adsorption measurements, employing the BET method at 77 K (BET BELSORP Mini II). A UV-visible spectrophotometer (JASCO V-570) was also used to measure dye concentrations in both feed and permeate solutions at the maximum absorbance wavelengths of MB and MO.

## Data availability

The data supporting this article have been included as part of the ESI.†

## Author contributions

Conceptualization and methodology: AA. Writing – original draft: ZB. Data curation and data analysis ZM.

## Conflicts of interest

There are no conflicts to declare.

## Supplementary Material

RA-014-D4RA04555F-s001
