# Supplementary material for: A simple, efficient, and rapid method for dye removal from wastewater using an IDA-GO@Fe3O4 magnetic nanocomposite
Source: RSC Adv. 2024 Sep 2;14(38):27843–51. doi: 10.1039/d4ra04555f (PMC11367389; doi:10.1039/d4ra04555f)
Supplement: RA-014-D4RA04555F-s001 [file RA-014-D4RA04555F-s001.pdf]

Supplementary Information (SI) for RSC Advances.
This journal is © The Royal Society of Chemistry 2024

# A simple, Efficient, and Rapid Method for Dyes removal from Wastewater using IDA-GO@Fe3O4 Magnetic Nanocomposite

Amir Abdolmaleki,*[a] Zahra Mohamadi [b] and Zahra Bazyar [c]

[a*] Department of Chemistry, College of Sciences, Shiraz University, Shiraz 71467-13565, I. R. Iran

[b] Department of Chemistry, Isfahan University of Technology, Isfahan 84156-83111, I. R. Iran

[c]Department of Chemistry, Behbahan Khatam Alanbia University of Technology, Behbahan, 63616-63973, I. R. Iran

*Corresponding author. E-mails : abdolmaleki@shirazu.ac.ir, abdolmaleki@ iut.ac.ir; Tel: +98-71-3613-7187; Fax: +98-71-3613-7310.

FTIR spectroscopy of GO, EDA-GO, and IDA-GO were reported by our group previously.[1] Briefly, the broad band at approximately 3320 $cm^{-1}$ (the O-H stretching vibration), the band at around 1732 $cm^{-1}$ (stretching vibration of the C=O bond of Carboxylic acid groups), and bands at 1224, and 1066 $cm^{-1}$ (stretching of C-OH, and of C-O bond, respectively) confirms the GO synthesis. After modification with EDA, a new band at 1656 $cm^{-1}$ (C=O bond of amide groups and additional bands attributed to the asymmetric stretching vibrations of C-N bonds, as well as the out-of-plane vibrations of $NH_2$ and NH, and the stretching vibrations of N-H confirms the structure. In the IDA-GO spectrum, a prominent band at 1743 $cm^{-1}$ indicates the presence of carboxylic acid groups. The absence of the 1547 $cm^{-1}$ band confirms the successful covalent bonding of acetic acid groups to EDA-GO and the transformation of primary amine groups into tertiary ones.

Also, EDA-GO and IDA-GO Raman spectra upon functionalizing graphene oxide with ethylenediamine (EDA-GO), the D and G peaks are observed at 1349 and 1584 $cm^{-1}$, respectively. The D/G ratio increases to 1.06, indicating the incorporation of $sp^3$ hybridized carbon species onto the graphene oxide sheets. The D/G ratio for IDA-GO is 1.05, with D and G peaks at 1344 and 1576 $cm^{-1}$, respectively. This suggests suitable oxidation and successful functionalization of graphene oxide. [1]

TGA analysis was reported in our previous report. [1] Briefly, the GO thermogram displays two distinct stages of weight loss, the first stage, corresponds to the degradation of $CO_2$-containing groups. The second stage, is attributed to the pyrolysis of the carbonaceous framework. In the EDA-GO thermogram, weight reduction begins at 200 °C and continues up to 800 °C. This gradual weight loss indicates an increase in hydrogen bonding between the functional groups in G-$NH_2$ compared to GO, due to the functionalization of graphene oxide sheets. Consequently, the remaining weight of EDA-GO is higher than that of GO. The IDA-GO thermogram demonstrates the pyrolysis of ethylenediamine and carboxylic acid groups at temperatures between 210 and 340 °C. Additionally, weight loss occurs between 340 and 450 °C due to the decomposition of the carbonaceous framework and the removal of organic groups from the IDA-GO surface. [1]

**Table S1** Thermodynamic parameters for MB and MO absorbed by IDA-GO/$Fe_3O_4$

| | Freundlich | | | Langmuir | | |
|---|---|---|---|---|---|---|
| | *n* | $K_f$ | $R^2$ | $K_L$(l/mg) | $R_L$ | $R^2$ |
| **MB** | 9.40 | 260.20 | 0.7318 | 8.00 | $4.40\times10^{-4}$ | 0.99<br>60 |
| **MO** | 3.40 | 45.80 | 0.8973 | 0.30 | $2.50\times10^{-2}$ | 0.99<br>44 |

**Table S2 Pseudofirst-order and pseudosecond-order kinetic parameters**

| | Pseudo-first-order model | | | | Pseudo- second -order model | | | |
|---|---|---|---|---|---|---|---|---|
| | $q_{e\ (exp)}$ | $q_{e\ (cal)}$ | $K_1$ | $R^2$ | $q_{e(exp)}$ | $q_{e(cal)}$ | $K_2$ | $R^2$ |
| **MB** | 200.00 | 35.40 | $1.60\times10^{-2}$ | 0.9399 | 200.00 | 200.00 | $9.8\times10^{-4}$ | 0.9999 |
| **MO** | 98.00 | 67.10 | $1.50\times10^{-2}$ | 0.8834 | 98.00 | 105.20 | $3.6\times10^{-4}$ | 0.9984 |

## Reference


1 A. abdolmaleki, z. mohamadi, H. Fashandi, Z. Bazyar, Chemical Engineering Journal, 2024,**481**, 148622.
